# Predictive Value of the Inflammatory Burden Index for Pathological Complete Response in HER2-Positive and Triple-Negative Breast Cancer Receiving Neoadjuvant Chemotherapy: A Comparative Analysis with Conventional Inflammatory Indices

**DOI:** 10.3390/jcm15176500

**Published:** 2026-08-22

**Authors:** Merve Turan, Özge Demirkıran

**Affiliations:** Department of Medical Oncology, Faculty of Medicine, Aydın Adnan Menderes University, 09010 Aydın, Türkiye; ozgedemrkrn@gmail.com

**Keywords:** inflammatory burden index, pathological complete response, neoadjuvant chemotherapy, breast cancer, HER2-positive, triple-negative, C-reactive protein, neutrophil-to-lymphocyte ratio, dynamic biomarker, Ki-67

## Abstract

**Background/Objectives:** The inflammatory burden index (IBI), calculated as C-reactive protein (CRP) multiplied by the neutrophil-to-lymphocyte ratio (NLR), has demonstrated prognostic value across several solid tumors. Its role in breast cancer, however, has not been investigated. This study evaluated whether pretreatment or post-treatment IBI could predict pathological complete response (pCR) in patients with HER2-positive or triple-negative breast cancer (TNBC) receiving neoadjuvant chemotherapy (NAC). **Methods:** This single-center retrospective study included 61 patients who completed NAC followed by surgery between 2019 and 2025. IBI was calculated before and after NAC, and the treatment-related change (ΔIBI) was assessed. Conventional inflammatory indices, including NLR, platelet-to-lymphocyte ratio (PLR), lymphocyte-to-monocyte ratio (LMR), systemic immune-inflammation index (SII), systemic inflammation response index (SIRI), C-reactive protein-to-albumin ratio (CAR), and absolute lymphocyte count (ALC), were evaluated for comparison. Analyses included Mann–Whitney U tests, paired Wilcoxon signed-rank tests, receiver operating characteristic (ROC) curve analysis, and multivariable logistic regression. **Results:** Twenty-nine patients (47.5%) achieved pCR. No pretreatment or post-treatment inflammatory index was significantly associated with pCR. In paired within-patient analysis, IBI increased significantly during treatment only in patients achieving pCR (*p* = 0.036), while remaining unchanged in the non-pCR group (*p* = 0.627). CAR showed an identical pattern, increasing exclusively in the pCR group (*p* = 0.013). This selective rise was not observed for any index lacking a CRP component and was independent of molecular subtype, anti-HER2 therapy, and chemotherapy regimen. On ROC analysis, ΔCAR yielded the highest area under the curve (AUC) among all inflammatory indices (0.637; *p* = 0.067), followed by ΔIBI (0.606; *p* = 0.157); neither reached statistical significance. Ki-67 was the only independent predictor of pCR (AUC 0.724; *p* = 0.003; optimal cutoff ≥25%). **Conclusions:** This is the first study to evaluate IBI in HER2-positive and TNBC receiving NAC. Static IBI values did not predict pCR. The selective rise in IBI and CAR during treatment in patients achieving pCR—two independently formulated CRP-based indices showing an identical pattern—suggests that the CRP component carries the biologically relevant signal. This hypothesis-generating observation warrants prospective validation in larger cohorts.

## 1. Introduction

Breast cancer remains the most frequently diagnosed malignancy among women worldwide, with approximately 2.4 million new cases and 694,000 deaths reported globally in 2024 [1]. Neoadjuvant chemotherapy (NAC) is now the standard approach for locally advanced HER2-positive and triple-negative breast cancer (TNBC). It downstages the tumor to improve surgical outcomes and also allows in vivo chemosensitivity to be assessed directly [2]. In contemporary practice, neoadjuvant approaches for HER2-positive breast cancer include both anthracycline-taxane-based regimens with dual HER2 blockade (trastuzumab plus pertuzumab) and carboplatin-containing combinations (TCbHP), both of which achieve pCR rates exceeding 50–60% in pivotal trials [3]. For TNBC, the addition of pembrolizumab to standard chemotherapy has significantly improved pCR rates (64.8% vs. 51.2%) and has become a new standard of care in eligible patients [4]. Pathological complete response (pCR), defined as the absence of residual invasive disease in the breast and axillary lymph nodes (ypT0/is ypN0), is the most clinically meaningful surrogate endpoint after NAC, particularly in these aggressive molecular subtypes [5]. Patients who achieve pCR have markedly better event-free and overall survival than those with residual disease, with risk reductions above 75% reported in TNBC cohorts [6]. Identifying reliable pretreatment and on-treatment biomarkers that predict pCR has therefore become a major aim of translational breast cancer research, with implications for treatment intensification, de-escalation, and patient counseling [2].

Over the past decade, several peripheral blood-derived inflammatory indices have been investigated as accessible, low-cost biomarkers of NAC response. The neutrophil-to-lymphocyte ratio (NLR), platelet-to-lymphocyte ratio (PLR), lymphocyte-to-monocyte ratio (LMR), systemic immune-inflammation index (SII), and systemic inflammation response index (SIRI) are the most extensively studied; they reflect the balance between pro-tumorigenic innate immunity and anti-tumor lymphocytic immunity. Their predictive value for pCR, however, remains inconsistent: a recent meta-analysis found that low pretreatment NLR was associated with pCR, whereas PLR, LMR, SII, and SIRI showed no consistent association [7], and several subtype-specific cohorts in HER2-positive, triple-negative, and luminal breast cancer populations receiving NAC have likewise failed to confirm a consistent predictive role for these latter indices [8,9,10].

A composite biomarker that explicitly incorporates an acute-phase reactant may address this limitation. The inflammatory burden index (IBI), calculated as C-reactive protein (CRP) multiplied by the neutrophil-to-lymphocyte ratio, was developed in a large multicenter cohort and outperformed conventional inflammatory indices in predicting survival across several cancer types, including non-small cell lung, colorectal, esophageal, and gastric cancer [11,12,13,14]. It has also shown prognostic value in the neoadjuvant setting specifically [15,16].

Despite this evidence, IBI has not yet been evaluated in patients undergoing NAC for breast cancer, even though preliminary data suggest that other CRP-containing composite indices may outperform conventional cellular ratios in this setting [8,9]. Because post-treatment inflammatory markers have shown greater predictive value than pretreatment measurements in several breast cancer studies [17,18], measuring IBI at both time points may better define its predictive role. We therefore aimed to evaluate the independent predictive value of pretreatment (pre-NAC) and post-treatment (post-NAC) IBI for pCR in patients with HER2-positive and triple-negative breast cancer.

## 2. Materials and Methods

### 2.1. Study Design and Population

**Figure 1:**
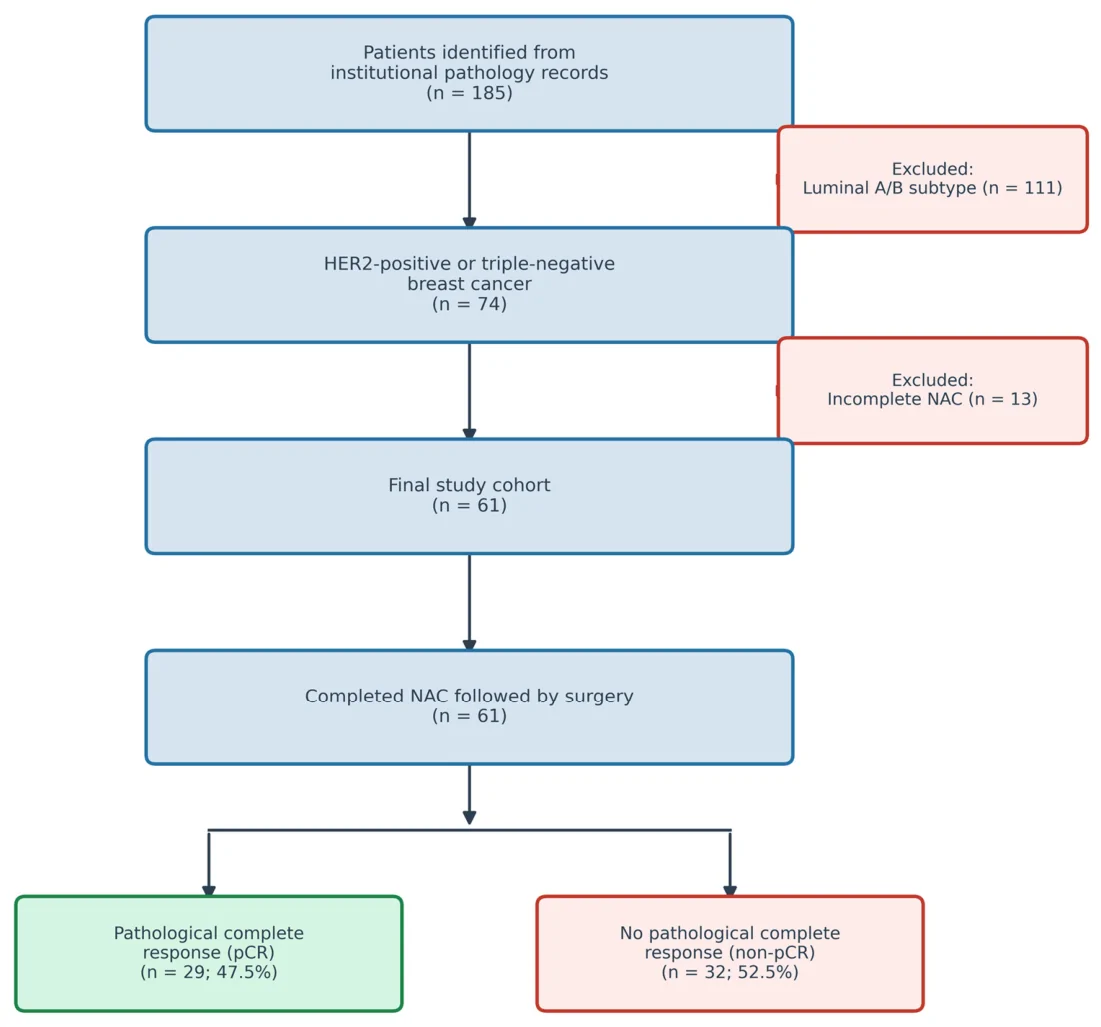
Patient selection flow diagram.

This single-center, retrospective study was conducted at the Department of Medical Oncology, Aydın Adnan Menderes University Faculty of Medicine, Türkiye. We reviewed the medical records of patients with histopathologically confirmed HER2-positive or triple-negative breast cancer who received NAC followed by definitive surgery between January 2019 and December 2025. Initially, 185 patients were identified from institutional pathology records. After excluding 111 patients with luminal A or B subtypes, 74 patients remained. An additional 13 patients were excluded due to incomplete NAC regimens. Consequently, 61 patients met all eligibility criteria and formed the final study cohort (Figure 1).

#### 2.1.1. Inclusion Criteria

Patients were eligible for inclusion if they met all of the following criteria: age ≥ 18 years; histopathologically confirmed HER2-positive or triple-negative breast cancer; receipt of NAC followed by surgery; availability of complete blood count and CRP values obtained at least two weeks after the last NAC cycle and within four weeks before surgery; and availability of a surgical pathology report.

#### 2.1.2. Exclusion Criteria

Patients were excluded if they had an active infection or autoimmune disease at the time of blood sampling, a concurrent active malignancy, or laboratory data insufficient to calculate pre-NAC or post-NAC IBI. Patients who did not complete NAC or for whom surgical pathology results were unavailable were excluded from the analysis.

### 2.2. Data Collection and Inflammatory Index Calculation

Patient demographics, tumor characteristics, treatment regimens, and laboratory values were retrieved from the institutional hospital information system. All patients received anthracycline-based chemotherapy (AC or EC) followed by a taxane, with dual HER2 blockade (trastuzumab and pertuzumab) added for HER2-positive patients. All included patients completed their planned NAC regimen. Detailed treatment characteristics are presented in Table 1. Pretreatment (pre-NAC) complete blood count and CRP values were obtained within one week before the initiation of NAC. Post-treatment (post-NAC) complete blood count and CRP values were obtained at least two weeks after the last cycle of NAC and within four weeks before surgery.

The inflammatory burden index (IBI) was calculated separately for both time points using the following formula:

IBI = CRP (mg/dL) × Neutrophil count (×10^3^/µL)/Lymphocyte count (×10^3^/µL)

The change in IBI over the course of treatment (ΔIBI) was calculated as:

ΔIBI = Post-NAC IBI − Pre-NAC IBI

From the same blood samples, the following additional inflammatory indices were also calculated for comparison: NLR, PLR, LMR, SII (defined as platelet count × neutrophil count/lymphocyte count), SIRI (defined as neutrophil count × monocyte count/lymphocyte count), and C-reactive protein-to-albumin ratio (CAR, defined as CRP/albumin) [7,11,19]. Absolute lymphocyte count (ALC) was also recorded as a direct measure of host immune status. For each index, the change during treatment (Δ) was calculated as the post-NAC value minus the pre-NAC value.

### 2.3. Pathological Assessment

Surgical pathology reports were reviewed to determine treatment response following NAC. Pathological complete response (pCR) was defined as the absence of residual invasive carcinoma in the breast and axillary lymph nodes (ypT0/is ypN0), which carried the strongest survival benefit in previous pooled analyses [5]. Patients not meeting this definition were classified as having residual disease (non-pCR). Tumor histological subtype, grade, and HER2/hormone receptor status were determined according to institutional pathology protocols based on immunohistochemistry and, where applicable, in situ hybridization testing. HER2-positive breast cancer was defined as HER2 3+ on immunohistochemistry or HER2 2+ with confirmatory amplification on in situ hybridization. Triple-negative breast cancer (TNBC) was defined as the absence of estrogen receptor (ER), progesterone receptor (PR), and HER2 expression (ER < 1%, PR < 1%, HER2 non-amplified), in accordance with current international consensus guidelines [20].

### 2.4. Statistical Analysis

Continuous variables were tested for normality using the Shapiro–Wilk test and are presented as mean ± standard deviation or median (interquartile range), as appropriate. Categorical variables are presented as frequencies and percentages and were compared using the chi-square or Fisher’s exact test, as appropriate. Between-group comparisons of continuous variables were performed using the independent-samples Mann–Whitney U test.

Receiver operating characteristic (ROC) curve analysis and the Youden index were used to determine optimal cutoff values for all continuous inflammatory indices and Ki-67. Given that only Ki-67 reached statistical significance on ROC analysis, cutoff-based interpretation is reported only for Ki-67; for the remaining indices, AUC values and their 95% confidence intervals and *p*-values are reported without cutoff specification. Patients were dichotomized into low and high Ki-67 groups based on the derived cutoff. The association between each IBI measurement and pCR was first assessed using univariable logistic regression. Given the limited number of pCR events (*n* = 29), the multivariable model was deliberately restricted to a small, pre-specified set of predictors to respect an events-per-variable ratio of approximately 10. Three variables were entered: Ki-67, the only baseline variable significantly associated with pCR; ΔIBI, the primary inflammatory variable of interest; and molecular subtype (HER2-positive vs. triple-negative), included as a clinically relevant adjustment factor. This corresponded to an events-per-variable ratio of approximately 9.7. Model performance was assessed using the omnibus test, the Hosmer-Lemeshow goodness-of-fit test, and the Nagelkerke R^2^.

The predictive performance of IBI was compared with that of NLR, PLR, LMR, and SII using ROC-AUC analysis, with pairwise comparisons performed using the DeLong test.

A two-sided *p*-value < 0.05 was considered statistically significant. All statistical analyses were performed using SPSS software (version 26.0; IBM Corp., Armonk, NY, USA); ROC curves were plotted in Python (version 3.11) using the Matplotlib (version 3.8) library from coordinates generated in SPSS.

### 2.5. Ethical Approval

This study was conducted in accordance with the principles of the Declaration of Helsinki and approved by the Ethics Committee for Non-Interventional Clinical Research, Faculty of Medicine, Aydın Adnan Menderes University (Decision No: 17; Protocol No: 2026/204). Given the retrospective nature of the study, the requirement for informed consent was waived. All patient data were anonymized prior to analysis.

## 3. Results

### 3.1. Patient Characteristics

A total of 61 patients who completed NAC and underwent definitive surgery were included in the analysis. The median age was 54.0 years (IQR: 47.0–60.0), and the mean body mass index was 26.1 ± 3.3 kg/m^2^. Thirty-two patients (52.5%) were postmenopausal. The cohort comprised 46 HER2-positive (75.4%) and 15 triple-negative (24.6%) tumors. Forty-eight patients (78.7%) received trastuzumab plus pertuzumab as anti-HER2 therapy. Twenty-nine patients (47.5%) achieved pCR (ypT0/is ypN0), and 32 (52.5%) had residual disease.

Baseline demographic and clinicopathological characteristics were well balanced between pCR and non-pCR groups. No significant differences were observed in age (*p* = 0.876), BMI (*p* = 0.515), menopausal status (*p* = 0.913), Eastern Cooperative Oncology Group (ECOG) performance status (*p* = 0.740), molecular subtype (*p* = 0.266), nodal status (*p* = 0.605), multifocality (*p* = 0.349), or anti-HER2 therapy (*p* = 0.255). Ki-67 proliferation index was significantly associated with pCR, with higher values observed in the pCR group (median 30.0% [IQR: 20.0–50.0] vs. 20.0% [IQR: 10.0–30.0]; *p* = 0.002). Among treatment characteristics, the AC regimen was associated with pCR more frequently than the EC regimen (72.4% vs. 27.6% in the pCR group; *p* = 0.024). Taxane type and total number of cycles did not differ between groups (Table 1). Post-neoadjuvant pathological staging (ypT and ypN) by response group is presented in Supplementary Table S1.

### 3.2. Comparison of Pre-NAC Inflammatory Indices Between pCR Groups

None of the pretreatment inflammatory indices differed significantly between pCR and non-pCR groups. Median pre-NAC IBI was 5.48 (IQR: 3.95–9.13) in the pCR group and 5.62 (IQR: 3.51–15.23) in the non-pCR group (*p* = 0.740). Similarly, pre-NAC NLR (*p* = 0.292), PLR (*p* = 0.977), LMR (*p* = 0.115), SII (*p* = 0.634), CAR (*p* = 0.065), SIRI (*p* = 0.254), and ALC (*p* = 0.121) showed no significant association with pCR (Table 2). The underlying raw hematological and biochemical values are provided in Supplementary Table S2. Among these, only pre-NAC albumin was marginally higher in the pCR group (median 4.50 vs. 4.30 g/dL; *p* = 0.048), whereas all other parameters were comparable between groups.

### 3.3. Comparison of Post-NAC Inflammatory Indices and Changes During Treatment (Δ) Between pCR Groups

Post-NAC inflammatory indices did not differ significantly between pCR and non-pCR groups. Median post-NAC IBI was 6.30 (IQR: 4.28–16.37) in the pCR group and 7.96 (IQR: 4.54–12.55) in the non-pCR group (*p* = 0.874). Post-NAC NLR (*p* = 0.697), PLR (*p* = 0.333), LMR (*p* = 0.386), SII (*p* = 0.194), CAR (*p* = 0.457), SIRI (*p* = 0.795), and ALC (*p* = 0.756) were also comparable between groups.

When changes during treatment were compared, ΔIBI showed the largest median between-group difference, with a median of 2.04 (IQR: −2.40 to 10.42) in the pCR group versus 0.50 (IQR: −3.58 to 3.04) in the non-pCR group, though this did not reach statistical significance (*p* = 0.157). ΔCAR showed a comparable trend (median 0.11 vs. 0.01; *p* = 0.067). ΔNLR (*p* = 0.157), ΔPLR (*p* = 0.603), ΔLMR (*p* = 0.525), ΔSII (*p* = 0.179), ΔSIRI (*p* = 0.214), and ΔALC (*p* = 0.378) similarly showed no significant differences between groups (Table 2).

### 3.4. Paired Analysis of Inflammatory Index Changes During Treatment

Paired Wilcoxon signed-rank tests were performed to assess within-patient changes in inflammatory indices from pre-NAC to post-NAC, stratified by pCR status.

In patients achieving pCR, IBI increased significantly during treatment (pre-NAC median 5.48 vs. post-NAC median 6.30; *p* = 0.036), whereas no significant change was observed in the non-pCR group (*p* = 0.627). A similar pattern was seen for CAR, which increased significantly during treatment only in the pCR group (*p* = 0.013) and not in the non-pCR group (*p* = 0.797). LMR decreased significantly in both the pCR (*p* = 0.004) and non-pCR groups (*p* = 0.002). PLR increased significantly only in the non-pCR group (*p* = 0.043). SIRI increased and ALC decreased significantly only in the non-pCR group (*p* = 0.047 and *p* = 0.001, respectively). NLR and SII did not change significantly in either group.

IBI and CAR were the only indices that increased significantly and exclusively in the pCR group. Both incorporate CRP, whereas none of the indices lacking a CRP component showed this pattern.

### 3.5. Confounder Analysis for ΔIBI

The change in IBI during treatment (ΔIBI) was not significantly associated with molecular subtype (*p* = 0.461), anti-HER2 therapy (*p* = 0.503), anthracycline regimen (*p* = 0.873), or taxane type (*p* = 0.208) (Supplementary Table S3).

### 3.6. ROC Analysis

**Figure 2:**
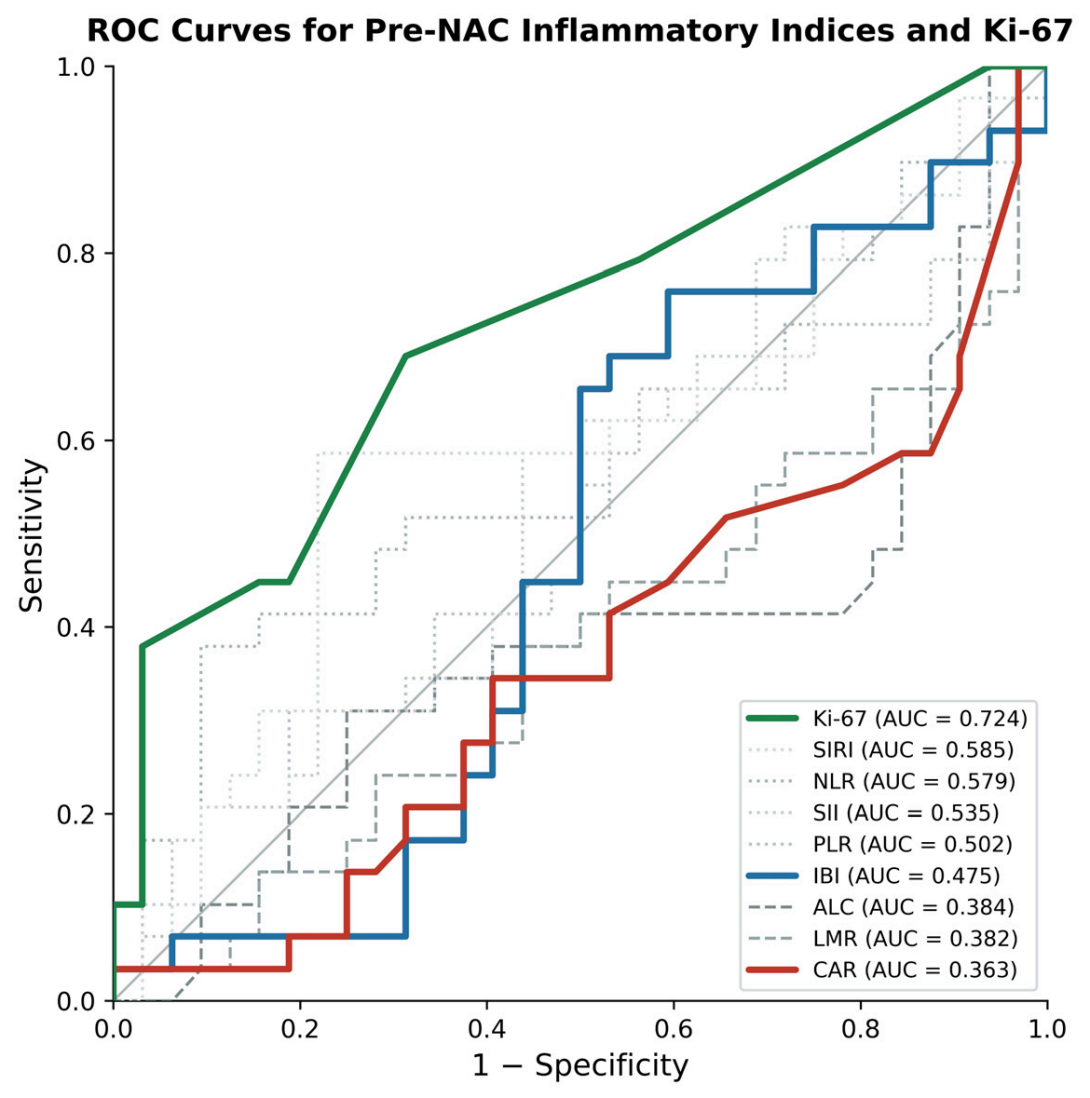
Receiver operating characteristic curves for pre-NAC inflammatory indices (IBI, CAR, NLR, PLR, LMR, SII, SIRI, ALC) and Ki-67 for predicting pathological complete response. Ki-67, CAR and IBI are highlighted in color; the other indices are shown in gray. CAR and IBI—the two indices central to our findings—are deliberately highlighted in distinct colors (red and blue), while the remaining indices are shown in grey to keep the visual focus on the key results. Each grey line is further distinguished by a different line style (solid, dashed, and dotted), and all indices are individually labeled with their AUC values in the legend.

**Figure 3:**
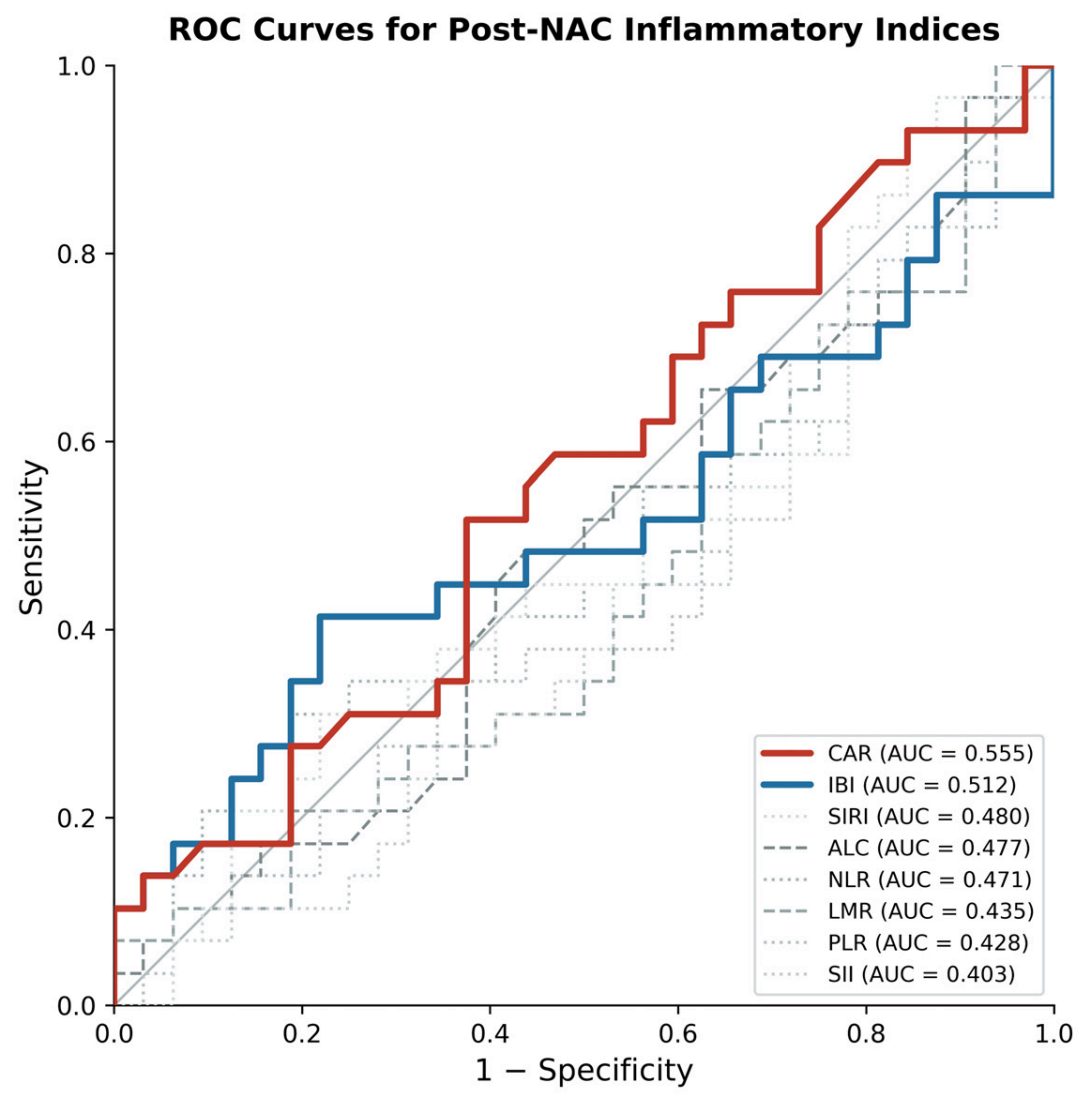
Receiver operating characteristic curves for post-NAC inflammatory indices (IBI, CAR, NLR, PLR, LMR, SII, SIRI, ALC) for predicting pathological complete response. CAR and IBI are highlighted in color; the other indices are shown in gray. CAR and IBI—the two indices central to our findings—are deliberately highlighted in distinct colors (red and blue), while the remaining indices are shown in grey to keep the visual focus on the key results. Each grey line is further distinguished by a different line style (solid, dashed, and dotted), and all indices are individually labeled with their AUC values in the legend.

**Figure 4:**
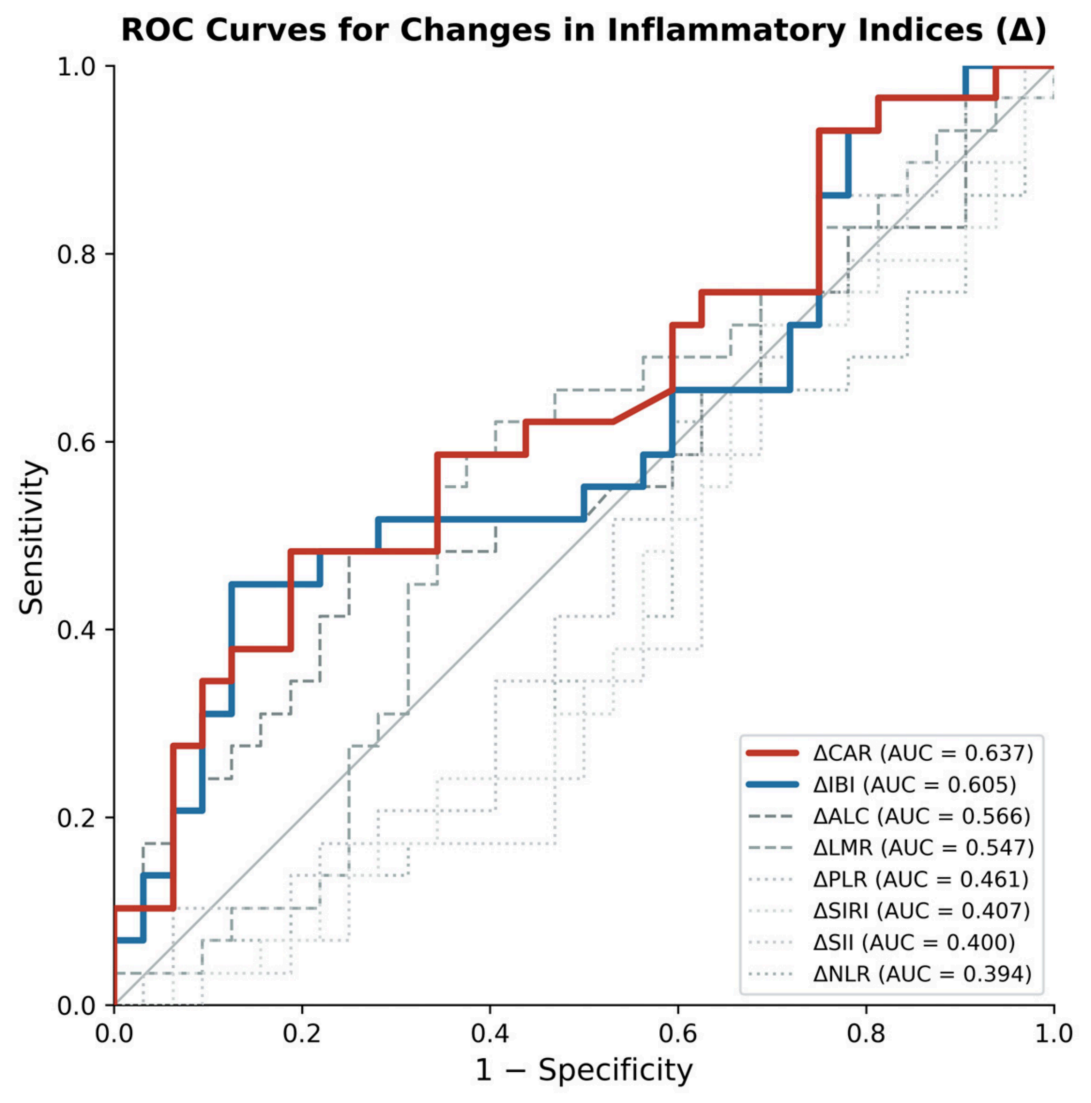
Receiver operating characteristic curves for the change (Δ) in inflammatory indices (IBI, CAR, NLR, PLR, LMR, SII, SIRI, ALC) during treatment for predicting pathological complete response. ΔCAR and ΔIBI are highlighted in color; the other indices are shown in gray. CAR and IBI—the two indices central to our findings—are deliberately highlighted in distinct colors (red and blue), while the remaining indices are shown in grey to keep the visual focus on the key results. Each grey line is further distinguished by a different line style (solid, dashed, and dotted), and all indices are individually labeled with their AUC values in the legend.

Receiver operating characteristic analysis was performed to evaluate the discriminatory performance of all inflammatory indices and Ki-67 for predicting pCR. Ki-67 was the only variable achieving a statistically significant AUC (0.724; 95% CI: 0.596–0.853; *p* = 0.003), with an optimal cut-off of ≥25% (sensitivity 69.0%, specificity 68.7%). Among the inflammatory indices, the two highest AUCs were obtained with the two CRP-based indices, ΔCAR (0.637; 95% CI: 0.496–0.778; *p* = 0.067) and ΔIBI (0.606; 95% CI: 0.460–0.751; *p* = 0.157), followed by pre-NAC NLR (0.579; *p* = 0.292) and ΔALC (0.566; *p* = 0.374). None of the inflammatory indices reached statistical significance (Table 3; Figure 2, Figure 3 and Figure 4).

The remaining indices, including ΔSIRI (0.407) and the pre-NAC and post-NAC forms of the other indices, showed AUC values at or near 0.500, indicating no discriminatory capacity for pCR prediction (Table 3).

To determine whether this signal was driven by the composite index or by its CRP component, CRP was analyzed separately. In the pCR group, the treatment-associated increase was confined to CRP (paired Wilcoxon *p* = 0.036), while no significant change was observed in the non-pCR group (*p* = 0.808) (Supplementary Table S2). ΔCRP and ΔIBI showed almost identical discriminatory performance (AUC 0.605 vs. 0.606), indicating that the signal was carried by the CRP component and that IBI did not add discriminatory value beyond CRP in this cohort.

### 3.7. Multivariable Analysis of Predictors of pCR

A multivariable binary logistic regression model was constructed to identify independent predictors of pCR, entering Ki-67, molecular subtype, and ΔIBI (Table 4). The model was statistically significant (omnibus χ^2^ = 16.40, *p* = 0.001) and showed adequate goodness of fit (Hosmer-Lemeshow *p* = 0.453; Nagelkerke R^2^ = 0.315), correctly classifying 68.9% of cases. Ki-67 was an independent predictor of pCR (OR 1.060 per 1% increase, 95% CI 1.015–1.106, *p* = 0.008). ΔIBI showed a borderline association that did not reach statistical significance (OR 1.060, 95% CI 0.998–1.127, *p* = 0.060), and molecular subtype was not significantly associated with pCR (OR 0.755, 95% CI 0.149–3.837, *p* = 0.735).

## 4. Discussion

This study examined whether the inflammatory burden index could predict pathological complete response in patients with HER2-positive or triple-negative breast cancer receiving NAC, and to our knowledge, it is the first to do so. Neither pretreatment nor post-treatment IBI was significantly associated with pCR, nor were the conventional inflammatory indices NLR, PLR, LMR, SII, SIRI, CAR, or ALC. Ki-67 was the only independent predictor of pCR (AUC 0.724; *p* = 0.003), consistent with recent reports from similar patient populations [8,10]. Although static IBI values did not predict pCR, paired within-patient analysis revealed a distinct dynamic pattern: IBI rose significantly during treatment only in patients achieving pCR (*p* = 0.036), while no change was observed in the non-pCR group (*p* = 0.627). This pattern was not seen for any other inflammatory index, and confounder analysis confirmed it was independent of molecular subtype, anti-HER2 therapy, and chemotherapy regimen. It should be emphasized that this within-patient change is not equivalent to predictive ability. In our data, no IBI measure, whether pre-NAC, post-NAC, or ΔIBI, discriminated between responders and non-responders on ROC analysis, and ΔIBI showed only weak, non-significant performance (AUC 0.606, *p* = 0.157). The paired increase in IBI among patients achieving pCR should therefore be read as a descriptive, hypothesis-generating observation rather than as evidence of clinical predictive utility.

The selective increase in IBI among patients who achieved pCR may be related to the inflammatory response caused by effective chemotherapy-induced tumor cell death. Unlike conventional cellular ratios, IBI includes CRP, which may explain why this change was detected only with IBI. CRP is not only a marker of inflammation; it has also been linked to tumor-related processes such as VEGF-mediated angiogenesis, NF-κB and PI3K/AKT signaling, and interactions with macrophages and monocytes in the tumor microenvironment [21]. Li et al. also showed that peripheral NLR was inversely associated with tumor-infiltrating lymphocytes and positively associated with tumor-associated macrophages in breast cancer specimens after NAC. Their findings support the idea that systemic inflammatory markers may reflect local immune changes within the tumor environment [22]. One possible explanation is that effective chemotherapy causes greater tumor necrosis, tissue injury, and release of damage-associated molecular patterns. This may lead to a temporary rise in CRP, which would be reflected in IBI but not necessarily in indices such as NLR or SII, since they do not include an acute-phase reactant. This is consistent with our finding that NLR alone did not change significantly in either group, while CRP multiplied by NLR, namely IBI, increased in patients with pCR. Separate analyses of the two components confirmed this: the treatment-associated change was carried by CRP, which rose significantly in the pCR group, while no significant change was observed in the non-pCR group (*p* = 0.808), and ΔCRP discriminated pCR almost identically to ΔIBI (AUC 0.605 vs. 0.606). In this cohort, therefore, IBI did not add discriminatory value beyond CRP alone. This most likely reflects the size of our sample: with 61 patients, the study was well placed to detect the dominant CRP effect but underpowered to capture any smaller additional contribution from the neutrophil-to-lymphocyte component of the index. Whether the composite adds value beyond CRP is thus a question that larger, multicenter studies will need to resolve, and the present findings may serve as a rationale for such work. Of note, the CRP rise occurred while neutrophil counts fell, a pattern that argues against a nonspecific inflammatory or infectious cause and is more compatible with a tumor-related acute-phase response. We emphasize that this interpretation is offered as a hypothesis rather than an established mechanism. Our study was not designed to test it: we did not measure CRP kinetics over time, nor did we relate the IBI change to imaging findings, to pathological necrosis in the surgical specimen, or to any translational marker of cell death. The mechanism underlying the observed association therefore remains unproven and should be seen as a direction for future work rather than a conclusion of the present study. The idea of a treatment-induced CRP rise as a marker of effective antitumor activity is not new. Milroy et al. showed in 1989 that CRP more than doubled during induction chemotherapy in chemosensitive but not non-responsive small cell lung cancer patients, attributing this to tumor necrosis provoking an acute-phase reaction [23], and similar CRP flare patterns have since been reported across tumor types treated with immune checkpoint inhibitors [24]. The selective IBI rise in our pCR patients may represent the NAC counterpart of this phenomenon. Supporting this interpretation, the CAR—a structurally distinct CRP-based index—showed an identical pattern: it increased significantly only in the pCR group (*p* = 0.013) and yielded the highest AUC among all inflammatory indices (ΔCAR 0.637). The convergence of two independently formulated CRP-based indices on the same signal reinforces the conclusion that the CRP component, rather than the cellular ratios, carries the biologically relevant information in this setting. In breast cancer, where baseline CRP is typically low, chemotherapy-induced tumor necrosis may produce a transient inflammatory surge that raises CRP—a pattern distinct from gastrointestinal malignancies with higher baseline inflammation, where effective chemotherapy instead lowers inflammatory markers [11,15].

IBI has shown consistent prognostic value across several solid tumors. In the original multicenter validation study of 6359 cancer patients, it outperformed all 16 competing inflammatory biomarkers for survival prediction [11]. Later studies found it superior to NLR and SII in colorectal cancer [13], esophageal squamous cell carcinoma [14], and non-small cell lung cancer [12]. In the neoadjuvant setting, Pelc et al. reported that IBI fell markedly after NAC in gastric cancer and independently predicted postoperative complications and survival [15], while Huang et al. found that IBI reached the highest concordance index among all inflammatory biomarkers for predicting tumor regression and survival in gastric cancer patients receiving neoadjuvant chemoimmunotherapy [16]. Our findings extend this evidence to breast cancer, with one important difference: in gastrointestinal malignancies static pretreatment IBI carries strong prognostic value, whereas in breast cancer the signal lies in its change over treatment rather than its absolute level.

This pattern fits a wider trend toward post-treatment and dynamic inflammatory markers in breast cancer. Wang et al. found that post-treatment NLR, PLR, LMR, and SII gave higher AUC values than their pretreatment counterparts for pCR prediction in 1994 patients [17]. Guirao García et al. similarly reported that post-neoadjuvant NLR and SII predicted pCR while baseline values did not [18]. Ozkan et al. showed that dynamic changes in immune-nutritional indices, particularly ΔGINI and NRI, outperformed static measurements for pCR prediction in a Turkish breast cancer cohort [25]. Our study adds IBI to these indices, where the treatment-induced change carries more biological information than the pretreatment snapshot.

Ki-67 was the only variable that independently predicted pCR in our cohort, with an optimal cut-off of ≥25% and an AUC of 0.724. This supports the view that tumor proliferation may be more closely related to chemotherapy response than systemic inflammatory markers in breast cancer. A similar finding was reported by Zihni et al., where Ki-67 was the only independent predictor of pCR, with an odds ratio of 7.08, while all inflammatory indices were nonsignificant [10]. Karatlı et al. also found Ki-67 to be an independent predictor of pCR in HER2-positive breast cancer, together with the CALLY index, using a similar cut-off of ≥27.5% [9]. Taken together, these findings suggest that highly proliferative tumors may be more sensitive to chemotherapy, whereas peripheral blood inflammatory ratios may not fully reflect this biological feature.

From a clinical standpoint, the fact that IBI changes selectively in patients who achieve pCR suggests that serial IBI monitoring during neoadjuvant treatment could act as an early, noninvasive marker of treatment efficacy. Unlike imaging-based response assessment or repeat biopsies, IBI comes entirely from routine blood tests (CRP and complete blood count) already drawn at regular intervals during chemotherapy, so it adds no cost or patient burden. The between-group difference in ΔIBI did not reach statistical significance in this small cohort. Still, the consistent direction of the effect and its independence from treatment-related confounders justify further study in larger prospective cohorts to test whether rising IBI during treatment can complement current response evaluation. Whether patients with a low or absent ΔIBI—who are more likely to have residual disease—should receive additional cycles or a different regimen is a question that prospective studies will need to answer. In current practice, pathological response is assessed only after surgery, leaving clinicians without real-time on-treatment guidance. This concept aligns with the growing interest in response-guided neoadjuvant therapy, where residual disease after standard chemotherapy may prompt escalation with agents such as capecitabine in TNBC or T-DM1 in HER2-positive disease. Whether IBI monitoring could contribute to such decisions remains to be tested, but the biological rationale and practical accessibility of the index make it a worthwhile candidate for prospective evaluation. Inflammatory indices have also been investigated as predictors of outcomes in patients receiving immune checkpoint inhibitors. A recent study demonstrated that elevated CRP, NLR, and SII independently predicted worse overall survival in ICI-treated patients across multiple tumor types, with CRP-containing models achieving the highest prognostic accuracy [26]. In breast cancer specifically, high NLR and CRP have been associated with reduced ICI response, likely reflecting an immunosuppressive tumor microenvironment, and indices such as SII and MLR are emerging as potential predictors of ICI benefit [27]. IBI has not yet been evaluated in this setting; however, given that CRP was the dominant signal in our cohort and that CRP-based models showed the strongest predictive performance in ICI-treated populations, the role of IBI alongside immune checkpoint inhibitor therapy—particularly in triple-negative breast cancer where pembrolizumab is increasingly incorporated into neoadjuvant protocols—warrants future investigation.

This study has several limitations. First, it was retrospective and conducted at a single center, so selection and information bias could not be fully avoided. Additionally, the six-year study period (2019–2025) during which neoadjuvant treatment strategies evolved may have introduced temporal bias, as patients treated in later years may have received more standardized or effective regimens. Although blood sampling was performed within predefined time windows before and after NAC, minor variations in sampling time within these windows could not be fully avoided and may have introduced some variability into the inflammatory indices. Second, the sample size was limited to 61 patients, which reduced the statistical power to detect small or moderate effects. For the same reason, the multivariable logistic regression model was limited to three predictors, corresponding to an events-per-variable ratio close to the minimum recommended threshold; the model should therefore be interpreted as exploratory. This may partly explain why ΔIBI, despite showing a numerically elevated AUC (0.606) among the inflammatory indices, did not reach statistical significance in either the ROC or multivariable analyses. Third, our cohort combined HER2-positive and triple-negative tumors, which differ in biology, treatment, and pCR rates, and most patients had HER2-positive disease (75.4%). Because the triple-negative group was small (*n* = 15), we analyzed the cohort as a whole and treated molecular subtype as a confounder rather than performing separate, adequately powered subtype analyses. Although ΔIBI did not differ by subtype and subtype was not an independent predictor of pCR, we cannot exclude subtype-specific effects, and our findings should not be extrapolated to either subtype individually without confirmation in larger, subtype-specific cohorts. Fourth, overall survival could not be analyzed because only one death occurred during follow-up. As a result, we could not assess the prognostic value of IBI in this setting, which should be addressed in future studies with longer follow-up and adequate numbers of survival events. Fifth, certain clinicopathological variables could not be fully assessed in this cohort. Histological grade was not consistently evaluable from core needle biopsy material across the study period. Tumor-infiltrating lymphocyte (TIL) was not included in the institutional pathology reporting protocol. Hormone receptor status among HER2-positive patients was not assessed as a primary study variable, as the study was designed around molecular subtype classification (HER2-positive vs. triple-negative) rather than hormone receptor co-expression; in TNBC, ER and PR negativity is inherent to the subtype definition, and the differential influence of hormone receptor status within the HER2-positive subgroup was outside the scope of this analysis. In addition, records of granulocyte colony-stimulating factor (G-CSF) administration were not consistently available in our retrospective dataset, so G-CSF use could not be included as a covariate. Post-NAC blood samples were, however, drawn at least two weeks after the last chemotherapy cycle, by which time the short-term effect of G-CSF on neutrophil counts has generally resolved, and consistent with this, the neutrophil-based indices (NLR and SII) did not rise after treatment in either group. A residual influence of supportive treatment on these indices nevertheless cannot be entirely excluded. We were also unable to assess TIL data, so we could not directly compare peripheral inflammatory markers with the local tumor immune microenvironment. Finally, we did not apply formal correction for multiple comparisons. Several inflammatory indices were examined across multiple time points and analyses, and all analyses other than the primary between-group comparison were exploratory. Consequently, the single significant paired result (the IBI increase in the pCR group, *p* = 0.036) may represent a type I error. We therefore regard this finding as hypothesis-generating rather than confirmatory, and it requires validation in an independent cohort.

## 5. Conclusions

This study is the first to evaluate IBI in patients with HER2-positive and triple-negative breast cancer receiving NAC. Static pretreatment and post-treatment IBI values, along with all conventional inflammatory indices examined, did not predict pCR. Ki-67 was the only independent predictor of pathological complete response. The selective rise in IBI during treatment in patients who achieved pCR, while it stayed unchanged in non-responders and independent of treatment-related confounders, represents a hypothesis-generating observation that warrants prospective validation. Notably, the CRP-to-albumin ratio (CAR)—a structurally distinct CRP-based index—showed an identical pattern and yielded the highest AUC among all inflammatory indices examined (ΔCAR 0.637, *p* = 0.067), reinforcing the conclusion that the CRP component carries the biologically relevant signal. This dynamic CRP-driven signal, absent for indices lacking a CRP component, should be tested in larger prospective multicenter cohorts with serial blood sampling and tumor microenvironment assessment. If validated, rising CRP-based indices such as IBI and CAR during NAC could serve as accessible, low-cost on-treatment indicators to identify patients at risk of non-pCR—potentially informing timely decisions about treatment intensification in the era of response-guided neoadjuvant therapy.

## Figures and Tables

**Table 1:** Baseline demographic and clinicopathological characteristics of the study population.

Variable	Total (*n* = 61)	pCR (*n* = 29)	Non-pCR (*n* = 32)	*p*
**Demographics**				
Age, years, median (IQR)	54.0 (47.0–60.0)	54.0 (47.0–59.0)	52.0 (46.0–60.8)	0.876
BMI, kg/m^2^, mean ± SD	26.1 ± 3.3	26.4 ± 3.4	25.8 ± 3.2	0.515
BMI category, *n* (%)				
Normal (18.5–24.9)	22 (36.1)	12 (41.4)	10 (31.3)	0.521
Overweight (25.0–29.9)	32 (52.5)	13 (44.8)	19 (59.4)	
Obese (≥30.0)	7 (11.5)	4 (13.8)	3 (9.4)	
Menopausal status, *n* (%)				
Premenopausal	29 (47.5)	14 (48.3)	15 (46.9)	0.913
Postmenopausal	32 (52.5)	15 (51.7)	17 (53.1)	
ECOG PS, *n* (%)				
0	35 (57.4)	16 (55.2)	19 (59.4)	0.740
1	26 (42.6)	13 (44.8)	13 (40.6)	
**Tumor Characteristics**				
Molecular subtype, *n* (%)				
HER2-positive	46 (75.4)	20 (69.0)	26 (81.3)	0.266
Triple-negative	15 (24.6)	9 (31.0)	6 (18.8)	
cT stage, *n* (%)				
cT1	11 (18.0)	6 (20.7)	5 (15.6)	0.864
cT2	46 (75.4)	21 (72.4)	25 (78.1)	
cT3	4 (6.6)	2 (6.9)	2 (6.3)	
cN stage, *n* (%)				
N0	15 (24.6)	8 (27.6)	7 (21.9)	0.467
N1	36 (59.0)	18 (62.1)	18 (56.3)	
N2–N3	10 (16.4)	3 (10.3)	7 (21.9)	
Nodal positivity, *n* (%)				
Negative	15 (24.6)	8 (27.6)	7 (21.9)	0.605
Positive	46 (75.4)	21 (72.4)	25 (78.1)	
Ki-67 (%), median (IQR)	20.0 (15.0–45.0)	30.0 (20.0–50.0)	20.0 (10.0–30.0)	0.002
Multifocality, *n* (%)				
No	45 (73.8)	23 (79.3)	22 (68.8)	0.349
Yes	16 (26.2)	6 (20.7)	10 (31.3)	
**Treatment**				
Anti-HER2 therapy, *n* (%)				
None	13 (21.3)	8 (27.6)	5 (15.6)	0.255
Trastuzumab + Pertuzumab	48 (78.7)	21 (72.4)	27 (84.4)	
Anthracycline regimen, *n* (%)				
AC	35 (57.4)	21 (72.4)	14 (43.8)	0.024
EC	26 (42.6)	8 (27.6)	18 (56.3)	
Taxane type, *n* (%)				
Paclitaxel	55 (90.2)	27 (93.1)	28 (87.5)	0.463
Docetaxel	6 (9.8)	2 (6.9)	4 (12.5)	
Total cycles, median (IQR)	16 (16–16)	16 (16–16)	16 (16–16)	-

Values are presented as median (IQR), mean ± SD, or *n* (%). *p*-values from independent samples *t*-tests (age, BMI), Mann–Whitney U tests (Ki-67), or chi-square/Fisher’s exact tests (categorical variables). A bold *p*-value indicates statistical significance (*p* < 0.05). Seven patients (11.5%) received carboplatin-containing regimens, and one received a pembrolizumab-containing regimen (KEYNOTE-522-based).

**Table 2:** Comparison of inflammatory indices between pCR and non-pCR groups.

Index	pCR (*n* = 29)	Non-pCR (*n* = 32)	*p* †
**Pre-NAC**			
IBI	5.48 (3.95–9.13)	5.62 (3.51–15.23)	0.740
CAR	0.49 (0.43–1.14)	0.57 (0.48–1.88)	0.065
NLR	2.02 (1.60–2.70)	1.83 (1.56–2.20)	0.292
PLR	112.57 (87.67–159.13)	111.85 (94.40–150.06)	0.977
LMR	4.24 (3.50–5.24)	4.58 (4.05–5.82)	0.115
SII	547.59 (401.37–808.16)	509.26 (364.04–783.19)	0.634
SIRI	1.20 (0.69–1.57)	0.94 (0.69–1.13)	0.254
ALC	2.05 (1.79–2.99)	2.50 (2.23–3.03)	0.121
**Post-NAC**			
IBI	6.30 (4.28–16.37)	7.96 (4.54–12.55)	0.874
CAR	1.13 (0.53–1.54)	0.96 (0.49–1.42)	0.457
NLR	1.95 (1.43–2.64)	2.11 (1.63–2.52)	0.697
PLR	127.88 (108.04–168.84)	145.80 (120.47–172.88)	0.333
LMR	3.35 (2.61–4.58)	3.74 (2.93–4.94)	0.386
SII	514.48 (403.02–745.16)	562.03 (426.34–871.46)	0.194
SIRI	1.20 (0.79–1.67)	1.29 (0.90–1.58)	0.795
ALC	2.10 (1.59–2.39)	2.10 (1.83–2.51)	0.756
**Δ (Post-NAC − Pre-NAC)**			
ΔIBI	2.04 (−2.40 to 10.42)	0.50 (−3.58 to 3.04)	0.157
ΔCAR	0.11 (−0.05 to 0.86)	0.01 (−0.32 to 0.21)	0.067
ΔNLR	−0.08 (−0.65 to 0.17)	0.12 (−0.45 to 0.66)	0.157
ΔPLR	13.29 (−13.41 to 37.43)	24.44 (−20.14 to 57.51)	0.603
ΔLMR	−0.49 (−1.87 to 0.05)	−0.94 (−2.31 to 0.27)	0.525
ΔSII	−30.32 (−232.22 to 92.80)	69.86 (−222.65 to 224.28)	0.179
ΔSIRI	0.10 (−0.28 to 0.38)	0.24 (−0.24 to 0.62)	0.214
ΔALC	−0.26 (−0.75 to 0.35)	−0.33 (−0.75 to −0.01)	0.378

Values are presented as median (interquartile range). † Mann–Whitney U test. IBI, inflammatory burden index; CAR, C-reactive protein-to-albumin ratio; NLR, neutrophil-to-lymphocyte ratio; PLR, platelet-to-lymphocyte ratio; LMR, lymphocyte-to-monocyte ratio; SII, systemic immune-inflammation index; SIRI, systemic inflammation response index; ALC, absolute lymphocyte count; NAC, neoadjuvant chemotherapy; Δ, change during treatment (post-NAC minus pre-NAC).

**Table 3:** Receiver operating characteristic analysis of inflammatory indices and Ki-67 for predicting pathological complete response.

Index	AUC (95% CI)	*p* †
Ki-67 (%)	0.724 (0.596–0.853)	0.003
ΔCAR	0.637 (0.496–0.778)	0.067
ΔIBI	0.606 (0.460–0.751)	0.157
Pre-NAC NLR	0.579 (0.430–0.727)	0.292
ΔALC	0.566 (0.419–0.714)	0.374
ΔLMR	0.547 (0.400–0.695)	0.525
Pre-NAC SII	0.536 (0.388–0.683)	0.634
Pre-NAC PLR	0.502 (0.353–0.651)	0.977
ΔSIRI	0.407 (0.264–0.551)	0.214

AUC, area under the curve; CI, confidence interval. For each index, the time point with the highest AUC is shown (Δ denotes the change from pre-NAC to post-NAC). † *p*-value tests the null hypothesis that AUC = 0.500. Ki-67 was the only variable with a statistically significant AUC; its optimal cut-off (Youden index) was ≥25% (sensitivity 69.0%, specificity 68.7%). Cut-off, sensitivity, and specificity are not reported for the remaining indices, as none reached statistical significance. IBI, inflammatory burden index; CAR, C-reactive protein-to-albumin ratio; NLR, neutrophil-to-lymphocyte ratio; LMR, lymphocyte-to-monocyte ratio; SII, systemic immune-inflammation index; PLR, platelet-to-lymphocyte ratio; ALC, absolute lymphocyte count; SIRI, systemic inflammation response index; NAC, neoadjuvant chemotherapy.

**Table 4:** Multivariable binary logistic regression for predictors of pathological complete response.

Variable	B	OR (Exp B)	95% CI	*p*
Ki-67 (%)	0.058	1.060	1.015–1.106	0.008
Molecular subtype (TNBC vs. HER2+)	−0.281	0.755	0.149–3.837	0.735
ΔIBI	0.059	1.060	0.998–1.127	0.060
Constant	−1.831	0.160	—	0.003

OR, odds ratio; CI, confidence interval; TNBC, triple-negative breast cancer; ΔIBI, change in inflammatory burden index during treatment. Model: omnibus χ^2^ = 16.40, *p* = 0.001; Nagelkerke R^2^ = 0.315; Hosmer-Lemeshow *p* = 0.453; overall classification accuracy 68.9%. Reference category for molecular subtype: HER2-positive.

## Data Availability

The data that support the findings of this study are available from the corresponding author upon reasonable request.

## Supplementary Materials

The following supporting information can be downloaded at: https://www.mdpi.com/article/10.3390/jcm15176500/s1, Table S1: Post-neoadjuvant pathological tumor (ypT) and nodal (ypN) staging by pCR status; Table S2: Raw hematological and biochemical parameters at pre-NAC and post-NAC time points, by pCR status; Table S3: Confounder analysis for the treatment-related change in IBI (ΔIBI).

## Abbreviations

ALC	Absolute lymphocyte count
AUC	Area under the curve
CAR	C-reactive protein-to-albumin ratio
CI	Confidence interval
CRP	C-reactive protein
ECOG	Eastern Cooperative Oncology Group
ER	Estrogen receptor
G-CSF	Granulocyte colony-stimulating factor
HER2	Human epidermal growth factor receptor 2
IBI	Inflammatory burden index
ICI	Immune checkpoint inhibitor
IHC	Immunohistochemistry
IQR	Interquartile range
LMR	Lymphocyte-to-monocyte ratio
NAC	Neoadjuvant chemotherapy
NLR	Neutrophil-to-lymphocyte ratio
OS	Overall survival
pCR	Pathological complete response
PLR	Platelet-to-lymphocyte ratio
PR	Progesterone receptor
ROC	Receiver operating characteristic
SII	Systemic immune-inflammation index
SIRI	Systemic inflammation response index
T-DM1	Trastuzumab emtansine
TIL	Tumor-infiltrating lymphocyte
TNBC	Triple-negative breast cancer
VEGF	Vascular endothelial growth factor
ypN	Pathological nodal stage after neoadjuvant therapy
ypT	Pathological tumor stage after neoadjuvant therapy
ΔIBI	Change in inflammatory burden index during treatment
