# Supplementary material for: Predictive Value of the Inflammatory Burden Index for Pathological Complete Response in HER2-Positive and Triple-Negative Breast Cancer Receiving Neoadjuvant Chemotherapy: A Comparative Analysis with Conventional Inflammatory Indices
Source: J Clin Med. 2026 Aug 22;15(17):6500. doi: 10.3390/jcm15176500 (PMC13565911; doi:10.3390/jcm15176500)
Supplement: Supplementary file 1 [file jcm-15-06500-s001.zip › Supplementary Table S1.pdf]

Supplementary Table S1. Post-neoadjuvant pathological tumor (ypT) and nodal (ypN) staging by pCR status.

Staging	pCR (n=29)	Non-pCR (n=32)	p
**ypT, n (%)**			
**ypT0**	29 (100.0)	2 (6.3)	<0.001
**ypT1**	0 (0.0)	19 (59.4)	
**ypT2**	0 (0.0)	11 (34.4)	
**ypN, n (%)**			
**ypN0**	29 (100.0)	19 (59.4)	0.001
**ypN1**	0 (0.0)	10 (31.3)	
**ypN2**	0 (0.0)	3 (9.4)	

Values are n (%). p-values from chi-square test. ypT and ypN were grouped (ypT1 includes 1, 1a, 1b, 1c; ypN1 includes 1, 1a, 1b, 1c; ypN2 includes 2a). By definition, all patients achieving pCR had ypT0 and ypN0. NAC, neoadjuvant chemotherapy.
