# Supplementary material for: Predictive Value of the Inflammatory Burden Index for Pathological Complete Response in HER2-Positive and Triple-Negative Breast Cancer Receiving Neoadjuvant Chemotherapy: A Comparative Analysis with Conventional Inflammatory Indices
Source: J Clin Med. 2026 Aug 22;15(17):6500. doi: 10.3390/jcm15176500 (PMC13565911; doi:10.3390/jcm15176500)
Supplement: Supplementary file 1 [file jcm-15-06500-s001.zip › Supplementary Table S3.pdf]

Supplementary Table S3. Confounder analysis for the treatment-related change in IBI (ΔIBI).

Factor	Comparison	Z	p
**Molecular subtype**	HER2-positive vs triple-negative	−0.737	0.461
**Anti-HER2 therapy**	Yes vs no	−0.669	0.503
**Anthracycline regimen**	AC vs EC	−0.160	0.873
**Taxane type**	Paclitaxel vs docetaxel	−1.259	0.208

ΔIBI, change in inflammatory burden index during treatment (post-NAC minus pre-NAC). p-values from Mann-Whitney U test. None of the factors was significantly associated with ΔIBI, indicating that the observed IBI dynamics were independent of molecular subtype and treatment characteristics.
