# Supplementary material for: Predictive Value of the Inflammatory Burden Index for Pathological Complete Response in HER2-Positive and Triple-Negative Breast Cancer Receiving Neoadjuvant Chemotherapy: A Comparative Analysis with Conventional Inflammatory Indices
Source: J Clin Med. 2026 Aug 22;15(17):6500. doi: 10.3390/jcm15176500 (PMC13565911; doi:10.3390/jcm15176500)
Supplement: Supplementary file 1 [file jcm-15-06500-s001.zip › Supplementary TableS2.pdf]

Supplementary Table S2. Raw hematological and biochemical parameters at pre-NAC and post-NAC time points, by pCR status.

Parameter	pCR (n=29)	Non-pCR (n=32)	p†	Paired p‡
**Pre-NAC**				
**Neutrophil (×10³/µL)**	5.00 (3.74–6.41)	4.74 (3.81–5.73)	0.697	—
**Lymphocyte (×10³/µL)**	2.05 (1.79–2.99)	2.50 (2.23–3.03)	0.121	—
**Monocyte (×10³/µL)**	0.51 (0.43–0.62)	0.50 (0.43–0.63)	1.000	—
**Platelet (×10³/µL)**	279 (220–328)	292 (256–363)	0.135	—
**CRP (mg/dL)**	2.00 (2.00–4.95)	2.30 (2.00–8.43)	0.314	—
**Albumin (g/dL)**	4.50 (4.30–4.60)	4.30 (4.20–4.50)	0.048	—
**Post-NAC**				
**Neutrophil (×10³/µL)**	4.33 (3.00–4.72)	4.26 (3.47–5.93)	0.214	—
**Lymphocyte (×10³/µL)**	2.10 (1.59–2.39)	2.10 (1.83–2.51)	0.756	—
**Monocyte (×10³/µL)**	0.58 (0.45–0.73)	0.62 (0.44–0.73)	0.800	—
**Platelet (×10³/µL)**	264 (218–333)	292 (257–335)	0.166	—
**CRP (mg/dL)**	4.40 (2.05–6.75)	4.00 (2.00–6.15)	0.392	0.036 / 0.808
**Albumin (g/dL)**	4.20 (4.05–4.55)	4.20 (3.93–4.40)	0.611	—

Values are presented as median (interquartile range). † Mann-Whitney U test (between groups). ‡ Paired Wilcoxon signed-rank test (pre-NAC vs post-NAC within each group); reported only for CRP as the key component driving the treatment-associated IBI signal (pCR group / non-pCR group). Neutrophil, lymphocyte, monocyte, and platelet counts are expressed as ×10³/µL; CRP as mg/dL; albumin as g/dL. Pre-NAC albumin was the only parameter significantly associated with pCR on between-group comparison (p=0.048); this was a marginal, exploratory finding not corrected for multiple comparisons. NAC, neoadjuvant chemotherapy; CRP, C-reactive protein; pCR, pathological complete response.
